# Treating Chronic Migraine With Neuromodulation: The Role of Neurophysiological Abnormalities and Maladaptive Plasticity

**DOI:** 10.3389/fphar.2019.00032

**Published:** 2019-02-05

**Authors:** Alessandro Viganò, Massimiliano Toscano, Francesca Puledda, Vittorio Di Piero

**Affiliations:** ^1^Headache Research Centre and Neurocritical Care Unit, Department of Human Neuroscience, Sapienza University of Rome, Rome, Italy; ^2^Molecular and Cellular Networks Lab, Department of Anatomy, Histology, Forensic Medicine and Orthopaedics, Sapienza University of Rome, Rome, Italy; ^3^Department of Neurology, Fatebenefratelli Hospital, Rome, Italy; ^4^Headache Group, Department of Basic and Clinical Neuroscience, King’s College Hospital, King’s College London, London, United Kingdom; ^5^University Consortium for Adaptive Disorders and Head Pain – UCADH, Pavia, Italy

**Keywords:** chronic migraine, plasticity, neuromodulation, NIBS, LTD, LTP, prophylaxis

## Abstract

Chronic migraine (CM) is the most disabling form of migraine, because pharmacological treatments have low efficacy and cumbersome side effects. New evidence has shown that migraine is primarily a disorder of brain plasticity and migraine chronification depends on a maladaptive process favoring the development of a brain state of hyperexcitability. Due to the ability to induce plastic changes in the brain, researchers started to look at Non-Invasive Brain Stimulation (NIBS) as a possible therapeutic option in migraine field. On one side, NIBS techniques induce changes of neural plasticity that outlast the period of the stimulation (a fundamental prerequisite of a prophylactic migraine treatment, concurrently they allow targeting neurophysiological abnormalities that contribute to the transition from episodic to CM. The action may thus influence not only the cortex but also brainstem and diencephalic structures. Plus, NIBS is not burdened by serious medication side effects and drug–drug interactions. Although the majority of the studies reported somewhat beneficial effects in migraine patients, no standard intervention has been defined. This may be due to methodological differences regarding the used techniques (e.g., transcranial magnetic stimulation, transcranial direct current stimulation), the brain regions chosen as targets, and the stimulation types (e.g., the use of inhibitory and excitatory stimulations on the basis of opposite rationales), and an intrinsic variability of stimulation effect. Hence, it is difficult to draw a conclusion on the real effect of neuromodulation in migraine. In this article, we first will review the definition and mechanisms of brain plasticity, some neurophysiological hallmarks of migraine, and migraine chronification-related (dys)plasticity. Secondly, we will review available results from therapeutic and physiological studies using neuromodulation in CM. Lastly we will discuss the results obtained in these preventive trials in the light of a possible effect on brain plasticity.

## Introduction

Chronic migraine (CM) (ICHD-III 1.3) (>15 days of headache per months, with >8 with migraine features for at least 3 months) affects about 2% of the general population and is the more disabling form of migraine, with a disability greater than that of episodic migraine (EM) (Dodick, 2006; Natoli et al., 2010).

Managing CM is extremely challenging for several reasons. First, only a few drugs, as OnabotulinumtoxinA, Topiramate, and Erenumab (the latter not available worldwide yet), have a clear level of evidence of efficacy (Silberstein et al., 2007, 2009; Tepper et al., 2017) Other available pharmacological options [as anticonvulsants (valproate), beta-blockers (atenolol and propranol), calcium antagonists (cinnarizine or flunarizine), anti-depressants (mostly tricyclic antidepressants)] or mini-invasive procedures [anesthetic Greater Occipital Nerve (GON) block], have in general a lower level of evidence (Saper et al., 2002; Spira et al., 2003; Yurekli et al., 2008; Magalhães et al., 2010; Sarchielli et al., 2014; Stovner et al., 2014; Inan et al., 2015; Cuadrado et al., 2017).

On average, the efficacy of pharmacological treatments does not exceed 50% of cases and the majority of these drugs are often poorly tolerated for their adverse effects (Evers et al., 2009; Blumenfeld et al., 2013). CM patients require more preventive lines and they annually spend more than episodic migraineurs, in medical expenses and loss of productivity (Blumenfeld et al., 2011; Berra et al., 2015).

There is thus a need of new more effective and better tolerated by patients pharmacological and non-pharmacological therapeutic options. To date, available non-pharmacological techniques include nutraceutical, ketogenic diet, cognitive-behavioral therapy, neurofeedback, psychotherapy, and Non-Invasive Brain Stimulation (NIBS). In particular, NIBS represents a very promising strategy for CM, since CM depends on a progressive maladaptation of the brain to sensory stimuli, and then it is theoretically possible reverting maladaptive plasticity to restore pre-chronicity status.

Non-Invasive Brain Stimulation techniques can act on neural plasticity by modifying brain excitability for periods outlasting the stimulation itself. This is a fundamental prerequisite of any valuable prophylactic treatment in migraine. Moreover, NIBS can directly aim at the migraine-related neurophysiological abnormalities, so that interventions may be planned on a precise pathophysiological rationale. Lastly, NIBS avoids cumbersome medication-related side effects and drug-drug interactions that limit the use of pharmacological therapies (Blumenfeld et al., 2013; Ansari and Ziad, 2016).

Up to date, several NIBS interventions have been tried with different results depending on different methodologies and techniques (e.g., transcranial magnetic stimulation, transcranial direct current stimulation), or protocols (as high-frequency and low-frequency), brain regions chosen as targets (e.g., primary vs. associative cortex), and stimulations types (e.g., the use of inhibitory and excitatory stimulations on the basis of opposite rationales). Aside to these, other therapeutic interventions have been tried with peripheral nerve stimulations, as trigeminal nerve stimulation and vagus nerve stimulation. Although these techniques that are not properly considered as NIBS, in this review, we will include some the results from these trials, since preclinical and human studies showed that their efficacy rely on the same plasticity-mediated mechanism (Pilurzi et al., 2016; Buell et al., 2018; Mertens et al., 2018; Meyers et al., 2018).

Due to their use in migraine field, in the present review, we will consider as NIBS single-pulse and repeated transcranial magnetic stimulation (sTMS or rTMS), as well as anodal and cathodal transcranial direct current stimulation (tDCS). As peripheral stimulations, we included stimulations directed to cranial nerves, i.e., Superficial Trigeminal Stimulation (STS), Greater Occipital Nerve Stimulation (GONS), and vagal nerve stimulation (VNS).

## Neural Plasticity and Its Relationship to Chronic Migraine

### Synaptic Plasticity

The notion of plasticity dates back over 50 years ago, when Hebb and co-workers observed increased learning skill in those rats reared as pets at home in respect to laboratory-raised counterparts. On this observation, they postulated that a morphological change somehow occurs in the brain of these animals (particularly at the level of synapses) in response to a change in the environment, producing brain remodeling (Brown and Milner, 2003) (see Table 1 for definition of different forms of plasticity).

**Table 1:** Definitions of plasticity.

**Synaptic plasticity:** a plasticity mechanism based on the strengthening of synapses between neurons to encode mnemonic traces. These synapses are in fact activated as an ensemble in processes of formation or recall of memory traces (e.g., mnesic engram, motor patterns, and pain).
**Homosynaptic plasticity:** plasticity phenomena occurring in the synapse that is firing. It relies on modifications in synaptic weights or in the number of receptors expressed in the synaptic cleft. The two cardinal mechanisms responsible for homosynaptic plasticity are long term depression (LTD) and long term potentiation (LTP).
**Heterosynaptic plasticity:** plasticity phenomena occurring in synapses different from the firing synapse. In general these changes involved near synapses in an opposite way compared to the stimulated one. For example, if the firing synapse is undergoing LTP, the other synapses tend to present LTD.
**Neuronal plasticity:** referred to plasticity changes occurring in neurons respect to plasticity in non-neuronal structures, as oligodendrocytes and axonal myelination degree.
**Anatomic plasticity:** plasticity mechanism depending on an anatomical correlate (e.g., reduction or increase of gray matter, changes of brain connectivity, dendric and axonal sprouting, rewiring after a lesion). It includes non-synaptic forms of plasticity relying on changes of intrinsic neural excitability after a structural modification, e.g., after a stroke the injured tissue becomes hyperexcitable.
**Dysplasticity:** indicates the maladaptive reshaping of brain connections, leading to abnormal, either diminished or increased plasticity. Dysplasticity has been advocated as cause of several chronic and progressive neurological diseases, as Alzheimer disease, Huntington disease, depression, and schizophrenia.

These changes were firstly described in the cortex and include growth of dendrites, axonal sprouting, synaptic membrane modifications, and also synaptogenesis, gliogenesis, and neurogenesis (Sanes and Donoghue, 2000; Ward and Frackowiak, 2006; Wieloch and Nikolich, 2006).

Plasticity adaptive morphological changes can occur in response to environmental experiences and challenges. They also can happen after brain injury, since damaged brain has the same molecular and cellular properties of healthy brain to induce neural plasticity. However, in pathological conditions, as brain damage (e.g., major stroke or migraine chronification), changes in brain excitability tend to be more pronounced, widespread, or also aberrant, compared to those of healthy brain (Schmidt et al., 2012; Brennan and Pietrobon, 2018). The core of synaptic plasticity is the reshaping of the excitatory-inhibitory balance, through modifications of synaptic weights occurring in both excitatory and inhibitory synapses. This adaptation mostly relies on specific patterns of activity of pre-synaptic and post-synaptic neurons (Froemke, 2015). The two well-known long-term synaptic mechanisms of plasticity are long-term depression (LTD) and long-term potentiation (LTP). LTP and LTD are mathematically predicted by the Bienenstock–Cooper–Munro (BCM) theory (Bienenstock et al., 1982). LTD refers to a progressive reduction of the responses, while LTP indicates an increase of responses of the post-synaptic neuron.

At the molecular level, LTD and LTP responses depend on the function of *N*-methyl-D-aspartate (NMDA) receptors, whose activation, in response to presynaptic input, induces a Ca^2+^ influx into the postsynaptic neuron. This leads to changes of the strength in the synapsis connecting the pre- and the postsynaptic neuron, by means of functional and structural remodeling (MacDermott et al., 1986). According to the BMC model, an infrequent presynaptic activity releases a low level of glutamate that activates mostly AMPA receptors, whereas metabotropic and NMDA receptors remain inactive. By contrast, following an intense presynaptic discharge, NMDA receptor is activated and synaptic weight changes (Gu, 2002; Froemke, 2015).

Aside from NMDA receptors, GABA_A_ and GABA_B_, metabotropic and AMPA glutamatergic receptors, acetylcholine (ACh), noradrenaline (NA), serotonin (5-HT), dopamine (DA), histamine (Hist), oxytocin (Oxt), and also adenosine receptors are also linked to LTP plasticity, since they may be regulated by their own neurotransmitters and increase glutamate or reduce GABA. So far, these modulatory transmitters play a permissive role in plasticity, in auditory, somatosensory, and visual cortex (Gu, 2002; Froemke, 2015).

Besides BCM theory, another model of plasticity is the spike-timing-dependent plasticity (STDP) principle (Huang et al., 2017). STDP also is linked to the glutamatergic synapses properties but plasticity process depends on the timing between the pre- and post-synaptic spike. In this model, in fact, the weight of the synaptic plasticity becomes stronger whether the presynaptic spike occurs before the post-synaptic one, and weaker if the postsynaptic spike precedes the presynaptic one. STDP mechanism also depends on the activity of NMDA receptors and, consequently, on modulation of the Ca^2+^ influx into the postsynaptic neuron (Froemke, 2015).

The STDP is the physiological basis of the concept of “metaplasticity.” Metaplasticity refers to the fact that synaptic plasticity can be modulated differently varying the pattern of stimulation, like delivering spikes in triplets or trains of few pulses repeated several times, or administrating two or more stimulations in sequence. In some cases, plasticity can also be reversed (then termed “reversal plasticity”) with adequate combination of stimulations.

In excitatory synapses, STDP produces LTP if spikes from the presynaptic neuron anticipate the ones from the postsynaptic neurons. By contrast, LTD occurs if postsynaptic neuron fires before the presynaptic one (Markram et al., 1997; Song et al., 2000; D’amour and Froemke, 2015). In inhibitory circuits, LTP or LTD can both occur, regardless which spike occurs first, if the two spikes happened within or outside a precise time interval (Vogels et al., 2011; D’amour and Froemke, 2015).

Plasticity can develop though either homosynaptic and heterosynaptic mechanisms, which generally coexist. Homosynaptic plasticity happens in a stimulated synapse, according to BMC or STDP model. During the stimulation of a synapsis, however, the inactive synapses of the same network can develop plastic forms of LTP or LTD, in order to counterbalance and minimize the change of weight occurring in the stimulated one (Song et al., 2000). The coupling of homosynaptic LTP and heterosynaptic LTD basically has the purpose of controlling the excitatory-inhibitory tone at long-range networks level (Stent, 1973).

In condition as sensitization, excitatory-inhibitory balance may be altered toward a progressive enhancement of LTP. Homosynaptic LTP may facilitate the occurrence of heterosynaptic LTP phenomena instead of LTD. Neurophysiologically, it corresponds to an increase in the amplitude of evoked potentials recorded in humans (van den Broeke et al., 2010) and it may cause an increase of nociceptive response to unmodified stimulation (Harvey and Svoboda, 2007). This phenomenon may be even stronger in pathological conditions, as migraine chronification.

### Migraine Pathophysiology: Cycling Excitability

Migraine is a disorder characterized by an altered sensory processing, as it has been unveiled by several electrophysiological and imaging studies (for reviews, see de Tommaso et al., 2014; Harriott and Schwedt, 2014; Goadsby et al., 2017). In episodic migraineurs, during the migraine cycle (the alternating periods of wellbeing and pain), the abnormal functioning of the brain fluctuates according to the particular moment of the cycle itself.

During the interictal phase, migrainous brain is characterized by a low level of preactivation in all sensory (e.g., visual, somatosensory, auditory, etc.) and associative cortices. Affected cortices respond to external repetitive stimulation with an initial low response (that may resemble hypoexcitability), followed by a progressive increase of neural activity, instead of a progressive reduction (i.e., habituation) as the stimulation continues (de Tommaso et al., 2014).

In healthy subjects, sensory stimulation generally evokes cerebral responses though a dual-process, involving both sensitization and habituation of responses (Groves and Thompson, 1970). When a sensory stimulation begins, the receiving cortex produces at first an increase of evoked responses due to the novelty of the stimulation (i.e., sensitization) and later (if stimuli persist unmodified), the responses decrement (i.e., habituation). This is independent from neural fatigue since if some unexpected event occurs, it provokes a sudden reappearance of the initial response (i.e., dishabituation) (Thompson and Spencer, 1966).

The pattern found in migraineurs is in line with this theory of a ceiling effect regulating habituation (Thompson and Spencer, 1966; Groves and Thompson, 1970). A lower preactivation level drives toward a delayed start of habituation process because the ceiling threshold to be activated is reached lately or not reached at all. This altered response may depend on a deficit of serotoninergic projections from the brainstem to the thalamus and then to cortex (Coppola et al., 2007b). Reduced excitatory inputs from the thalamus produce in the cortex a slowing of the natural oscillations: for instance the visual cortex shifts from alpha (8–12 Hz) to theta (4–7 Hz) range with a consequent impairment of GABAergic interneurons, resulting in an increase of the high-frequency activity in the boundaries of the slowed-down area (this phenomenon is called “the edge-effect”) (Llinás et al., 1999; De Ridder et al., 2015). In normal conditions, high frequency gamma oscillations occur only transiently and mediate the conscious perception of external stimuli by binding different cortical networks (Melloni et al., 2007). In migraine, gamma oscillations of the visual cortex are increased and do not habituate as in normal subjects. This leads to recruitment and activation of multiple networks of neurons at once during a stimulation and eventually to hyperactivity (Coppola et al., 2007a).

Since gamma activity is more energy-demanding than other brain rhythms (Nishida et al., 2008; Huchzermeyer et al., 2013), this may explain how a habituation deficit conducts to metabolic strain, and ultimately to a migraine attack. The lack of habituation is maximal in the days preceding the attack (Coppola et al., 2009).

On the other hand, during the attack, the lower level of preactivation (found in the interictal phase) rises to normal values, sensitization increases and habituation normalizes, eventually leading to a state of hyperexcitability, whose manifestation is central sensitization, i.e., the increase of the normal nociceptive sensitization. When sensitization is set, the nociceptive threshold lowers so that the perception of similar noxious stimulations is amplified (Woolf and Thompson, 1991). In migraine during an attack trigeminal ganglion and thalamus are sensitized (Burstein et al., 2010; Mathew, 2011).

Central sensitization has both clinical (allodynia) and neurophysiological correlates [ictal laser-evoked potentials (LEPs) responses (de Tommaso et al., 2005)], and lasts for the entire duration of the attack, slowly disappearing with a return to the interictal state. During an attack, the normal habituation seems to be restored via a compensatory enhancement of inhibitory activity driven by the hyperexcitability state (Conte et al., 2010; Cosentino et al., 2014). It is interesting to notice that several indices of cortical excitability vary with the time elapsed from the last attack: excitability of the motor cortex is low far from attack and become higher as the attack approaches (Cortese et al., 2017a). As well, intracortical lateral inhibition, a measure of activity of inhibitory interneurons, follows the same dynamics (Coppola et al., 2016).

### Migraine Chronification as Maladaptive Synaptic and Anatomical Plasticity

Migraine chronification is clinically related to the repetition of migraine attacks. The number of attacks is the main risk factor for chronification itself (Buchgreitz et al., 2006). In CM, outside an attack, the neurophysiological response to repeated stimuli is similar to the pattern found in episodic form during an attack: hyperexcitability, central sensitization and normal habituation (Ayzenberg et al., 2006; Chen et al., 2011, 2012; Mathew, 2011; Schoenen, 2011; Viganò et al., 2018). Interestingly, when patients are successfully treated and return to episodic migraine, the low preactivation and the lacking habituation reappear (Chen et al., 2011, 2012).

The mechanism of the shift from episodic to CM is not still completely elucidated, however, it may depend on a maladaptive response to environmental sensory stimuli, leading to pain sensitization in trigeminal-cervical complex, thalamus, and cortical sensory and associative areas.

Homosynaptic synaptic plasticity may play a significant role in migraine transition from episodic to chronic form. Structures of the central nervous system may show central sensitization show central sensitization when nociception is enhanced with an increase in membrane excitability, synaptic efficacy or a reduced inhibition (Woolf and Thompson, 1991). Experimental evidence showed that sensitization of nociceptive responses at a trigeminal level is mediated by a combination of heterosynaptic and homosynaptic plasticity that are also responsible for the spatial spread of enhanced responses in neighboring cutaneous territories (Woolf and Thompson, 1991; Ikeda et al., 2006; Luo et al., 2008).

Moreover, homosynaptic LTD of sensory terminals is responsible for habituation, based on the fact that short-term habituation and synaptic depression coexist and show similar kinetics of onset and decay (Christoffersen, 1997; Glanzman, 2009; Gover and Abrams, 2009).

During chronification, each attack induces activation of excitatory and inhibitory circuits. However, inhibitory circuits are differently affected from excitatory ones, since they show a higher and faster adaptation and a slower recovery to a repeated stimulation (Wehr and Zador, 2003; Kuhlman et al., 2013). A higher number of attacks may induce LTD of inhibitory synapses while excitatory synapses are preserved, leading to a progressive disinhibition of brain responses and then to the patter of hyperexcitability found in CM. This state has been called “a never-ending attack” (Schoenen, 2011).

As neurotransmitter, serotonin seems to be directly involved. In a recent paper of our group, we investigated electrophysiological patterns associated to transition from CM to EM after GON anesthetic block (Viganò et al., 2018). We found that during the recovery from chronic to episodic migraine, an early increase of the serotonin firing (within the 1st week after GON block) was found in patients who had clinical improvement in the following weeks. By contrast, patients who didn’t benefit from the treatment serotonin firing remained low. The size of the increase of serotonin firing was linearly correlated to the clinical improvement. Interestingly, habituation passed from normal (in CM condition) to lacking (when patients improved to EM). Since in EM high serotonin is associated to normal habituation and low serotonin to lacking habituation, that support the idea that normal habituation in CM depends on plastic tuning of synapses rather than solely on ceiling effect as in EM. In our paper, we defined it “pseudonormal” to stress the different mechanism. Serotonin seems able modulate the excitatory/inhibitory balance and metaplasticity, shifting the activity of neural circuits from inhibition to excitation, as already found in the hippocampus (Kemp and Manahan-Vaughan, 2005). We measured serotonergic firing by using the intensity dependence of auditory evoked potentials (IDAP) that is a measure of 5-HT1B receptors activity (Proietti-Cecchini et al., 1997; Juckel et al., 2008; Wutzler et al., 2008). 5-HT1B receptors are involved, together with others 5-HT receptors, in plastic adaptation in different brain regions (Hurley et al., 2008; Dölen et al., 2013; Barre et al., 2016; Carhart-Harris and Nutt, 2017; Zhou et al., 2019).

Besides synaptic modifications in CM, plasticity may originate also from anatomical restructuration of dendritic spines and axonal connection, as happens in brain injury model, where synchronous electrical hyperactivity following the brain insult can promote axonal sprouting, resulting similar to LTP (Wieloch and Nikolich, 2006).

Both cortical hyperexcitability and the axonal sprouting play a role in promoting the neuroanatomical plasticity and consolidate new neural networks in response to a change in the environment (Buchli and Schwab, 2005; Dancause et al., 2005).

At a microscopic level some studies found same brain structures may change their morphology due to pain presence. The first hint of anatomical plastic changes in migrainous brain came from the evidence of alterations in thalamic structure, measured by fractal anisotropy (FA), according to migraine cycle (Coppola et al., 2014). This result was confirmed by subsequent experiment in different phases of migraine cycle in EM (Coppola et al., 2015). The changes in FA has been attributed to a rework of neuronal connections and dendritic arborizations, suggesting that the number of local circuits could be increased during the attack and decreased interictally (Beaulieu, 2002). This result gave an interpretative basis to look at structural data in CM.

To date, different studies have reported contrasting results on local changes in gray matters, however, a common feature seems to be present in the majority of them. In CM, several brain areas involved in migraine pathophysiology showed a decrease of the gray matter volume (GMV). In a recent paper, Coppola et al. (2017) found that in CM patients gray matter is reduced in the temporal lobe pole and gyrus, amygdala, hippocampus, pallidum, and orbitofrontal cortex, and also in the visual cortex and cerebellum in comparison to healthy subjects. It is interesting to notice that the alterations were found predominantly in the left hemisphere. This is also supported by a previous study that found a decrease of the GMV in the amygdala, insula, cingulate cortex and medial frontal gyrus, although the difference was found only between chronic and episodic migraneurs (Valfrè et al., 2008). In the same study by Valfrè et al. (2008), migraineurs showed a reduced local GMV in right superior temporal gyrus, parietal operculum, right inferior frontal gyrus and left precentral gyrus compared to healthy subjects, although none of the latter regions have had a correlation with clinical outcome while areas highlighted only in CM did (Valfrè et al., 2008). Another study performed on patients with CM and medication overuse headache (MOH) showed a larger reduction of brain volumes in the orbitofrontal cortex and left middle occipital gyrus of patients with MOH (Lai et al., 2016).

Interestingly, some of these alterations are correlated to clinical parameters, such as the frequency of migraine attacks and the duration of the disorder (Valfrè et al., 2008; Coppola et al., 2017). A study by Bilgiç et al. (2016) also found a decrease of the size of the cerebellum and brainstem, without, however, a correlation to clinical features.

On the other hand, in contrast with previous results, some studies showed an increase of GMV in amygdala, putamen and left temporal pole/parahippocampus (Lai et al., 2016; Neeb et al., 2017).

## NIBS-Induced Plasticity

Transcranial magnetic stimulation (TMS) and transcranial direct current stimulation (tDCS) are the most common NIBS methods used to study and modulate cortical excitability in experimental settings investigating neural plasticity. They act on both synaptic and anatomic plasticity. Some of the effects obtained by the stimulation are achieved from changes in the neuronal structures, elicited by external electric (tDCS) or magnetic (TMS) fields, beside the fact that external electric field causes displacement of intracellular ions, thus altering the internal charge distribution and modifying the neuronal membrane potential (Ruffini et al., 2013). Repetitive magnetic stimulation (rMS) is known to elicit structural remodeling of dendritic spines by remodeling postsynaptic gephyrin scaffolds, in addition to modifying synaptic GABAergic strength (Lenz and Vlachos, 2016).

### TMS Protocols

Repeated TMS protocols are able to induce amplitude changes in motor evoked potentials (MEPs) similar to those expected following LTP in the glutamatergic synapses (Huang et al., 2017). According to the BCM model, stimulation trains at high frequency (10–20 Hz) are able to induce LTP, whereas stimulation trains with a low frequency (around 1 Hz) induce LTD (Bliss and Collingridge, 1993; Pascual-Leone et al., 1994; Chen et al., 1997). TMS can easily induce metaplasticity (Huang et al., 2017). Not varying frequency nor intensity, the effect of neuromodulation changes according to the pattern of stimuli administration, as happens in theta burst stimulation (Huang et al., 2011).

Theta burst stimulation (TBS) is based on bursts of 3 pulses (triplets) delivered at 50 Hz and separated by 200 ms intervals (trains of 3 pulses are delivered at 5 Hz). Two types of TBS have a good effect: the intermittent (iTBS) and the continuous (cTBS) theta burst stimulation. iTBS is made with train of a 2 s (30 pulses) every 10 s (600 pulses in total). In cTBS, 50 Hz triplets are repeated continuously for a 40 s (600 pulses in total) (Huang et al., 2005).

Metaplasticity can also be achieved by the combination of priming stimulation with a conditioning stimulation. For example, if the priming is excitatory and the conditioning stimulation is inhibitory the effect of the conditioning stimulation can be reverted to excitation. A similar metaplasticity-like effect has been found following quadripulse stimulation (QPS) (Hamada et al., 2008) and theta burst stimulation (TBS) (Murakami et al., 2012).

rTMS influences brain excitability on a target cortex as well as in distant regions belonging to the same networks varying the functional connectivity between long-range areas. The application of excitatory QPS on M1 decreased interhemispheric functional connectivity of the contralateral M1, whereas inhibitory QPS did the opposite (Watanabe et al., 2014). The same results were replicated with a minor extent on S1 or DLPFC.

As mechanism, NMDA Ca^2+^-channels involvement has been demonstrated for high frequency rTMs (Liu et al., 2017), theta burst stimulation (TBS) (Huang et al., 2007), quadripulse stimulation (QPS) (Tanaka et al., 2015), and paired associative stimulation (PAS) (Stefan et al., 2002).

### tDCS Protocols

The mechanism underlying tDCS plasticity seems to be mediated by *N*-methyl-D-aspartate (NMDA) and γ-aminobutyric acid type A (GABA) receptors. Anodal stimulation reduces GABA, whereas cathodal stimulation reduced both glutamatergic and GABA levels [for an exhaustive review see (Stagg and Nitsche, 2011)]. This result is supported by the notion that pharmacological blockage of NMDA abolishes tDCS after-effects, while NMDA agonists enhance them (Nitsche et al., 2003). Moreover, animal studies have confirmed the involvement of NMDA receptors and brain-derived neurotrophic factor (BDNF) for the long-term effects observed after anodal tDCS, and adenosine A1 receptors after cathodal tDCS (Ammann et al., 2016). In a PET study, anodal stimulation enhanced rCBF while cathodal induced a decrement of rCBF (Lang et al., 2005).

However, predicting the outcome of a tDCS protocol is not straightforward, since several parameters may influence the final effect (Horvath et al., 2015). The stronger evidence of an effect is available for MEPs, since almost the totality of studies found the anodal stimulation is excitatory and cathodal is inhibitory (Priori et al., 1998; Nitsche and Paulus, 2001; Nitsche et al., 2003, 2005). For a review, see (Horvath et al., 2015). However, outside of the motor cortex, the studies yielded contrasting results. Visual evoked potentials (VEPs) resulted enhanced after either anodal or cathodal stimulation (Antal et al., 2004; Accornero et al., 2007). In two recent sham-controlled TMS EEG experiments (Romero Lauro et al., 2014; Varoli et al., 2018), authors showed that anodal stimulation on posterior parietal cortex produce an immediate and sustained increase of cortical excitability not limited to the stimulated region, but spread through all the fronto-parietal network and bilaterally, while the same experiment with cathodal stimulation yielded no significant results. This different result was attributed to the network properties: networks with a low baseline activity can respond better to anodal stimulation than cathodal (in the latter stimulation may suffer from a flooring effect), while the opposite condition, namely that anodal stimulation may be less effective on brain regions with high baseline activity, i.e., ceiling effect, occurs rarely.

## Summary of Neuromodulation Techniques and Studies in Chronic Migraine

### Non-invasive Brain Stimulation (NIBS) Techniques

#### Transcranial Magnetic Stimulation (TMS)

Some rather surprising results in migraine prophylaxis have been obtained by sTMS (see Table 2). sTMS was firstly implemented in clinical trials as a non-pharmacological acute treatment for its ability to block cortical spreading depression in rats, as well as inhibiting the firing rate of nociceptive thalamocortical projection neurons (Andreou et al., 2016). However, a large United Kingdom post-market survey, performed on 190 migraineurs, using a hand-held sTMS device for acute headache relief (Bhola et al., 2015), showed that at 3 months, both episodic and CM groups (the latter constituting two thirds of the population) had a significant reduction in the number of headache days respect to baseline. Moreover, a similar study (ESPOUSE trial) evaluated sTMS treatment in both the acute and preventive setting have shown a reduction in headache frequency in both episodic and CM subjects (Starling et al., 2018).

**Table 2:** Summary of neuromodulation trials involving CM patients.

Authors	Study	Device	Participants	Stimulation protocol	Duration of the treatment (sessions)	Stimulated area	Results	Notes
Clarke et al., 2006	Open-label	sTMS	MA (*n* = 10). MoA (*n* = 25). pMO (*n* = 6). Up to headache frequency of 1/day (but no data on % of CM patients).	2 pulses (5 s apart)	1–3	MA: visual or somatosensory cortex. MoA: pain perceiving area.	Pain reduction Lower relapse rate the next day. Trend for a higher improvement in patients with more sessions.	No restriction on medications. (e.g., analgesics, narcotics, antiemetics, sedatives).
Bhola et al., 2015	Open-label	sTMS	MA+MoA (*n* = 59). CM (*n* = 131, 87 with also MOH).	1/2 pulses acutely	No limit within 12 weeks	V1	Pain reduction. Less headache days. Lower HIT-6 score.	No limitation to change medication during the TMS treatment. MOH was discouraged. Patients on preventives therapy (*n* = 64).
Starling et al., 2018 ESPOUSE trial	Open-label	sTMS	MA (*n* = 44). MoA (*n* = 88). CM (*n* = 13).	Prevention: 2 pulses repeated after 15 min interval twice/day. Acute: 3 pulses repeated after 15 min interval (up to 2 times).	Four pulses twice daily, 3 months of treatment		Less headache days. Higher complete responder rate. Reduce medication intake.	2.3% on prophylaxis. MOH excluded. Also excluded: mental impairment. Severe active major depression or major psychiatric illness Other neuromodulation therapy in the past month Onabotulinum toxin A in the past 4 months
Conforto et al., 2014	RCT, double blind, sham-controlled.		CM (*n* = 9).	High-frequency (10 Hz), 32 trains of 5 s, every 30 s of pause (at 110% of MT).	23 sessions over 8 weeks.	Left DLPFC.	Inferior to sham.	Excluded patients with concomitant depression.
Misra et al., 2013	RCT, sham-controlled	rTMS	CM (*n* = 100). Included MOH.	600 pulses in 10 trains at 10 Hz with 45.5 s of intertrain interval.	3/week for 1 month.	Primary motor cortex	Primary outcomes: >50% responders (headache frequency); >50% responders (pain). Secondary outcome: any improvement pain, functional disability, rescue medication, adverse events	
Shehata et al., 2016	RCT	rTMS	CM (*n* = 29).	20 trains of 100 stimuli at 10 Hz in tri-weekly sessions.	1 month.	Primary Motor cortex.		Comparison to botulinum toxin-A injections. Excluded: MOH, patients on prophylaxis (within 4 weeks), comorbid psychiatric disorders.
Rapinesi et al., 2016	RCT	Deep rTMS	Treatment-resistant CM, no MOH.	10 Hz trains of 600 pulses.	4 weeks	Lateral and medial part of the prefrontal cortex bilaterally.	Decrement in pain intensity, number of headache days and also depressive symptoms compared to the standard treatment.	Included patients with depression (*n* = 3) in both arms.
Teepker et al., 2010	RCT	rTMS	EM (*n* = 27).	Two trains of 500 pulses at 1 Hz, with inter-train interval 60 s.	12 in 5 weeks.	Vertex.	Reduced headache days. No different with sham.	–
Chen et al., 2016	Open-label clinical trial	cTBS	EM (*n* = 6) and CM (*n* = 3).	Bursts of 3 pulses at 50-Hz every 200-ms intervals for 40 s.		20 cTBS daily for 4 weeks.	Reduced headache days.	CM patients were on prophylaxis.
Antal et al., 2011	RCT, sham- controlled trial with crossover design.	Cathodal tDCS	CM (*n* = 13).	Cathodal 1 mA for 15 min once a day, every 2nd day.	3 days/week for 6 weeks	Occipital cortex.	Reduced intensity of pain (only superior to sham).	3 weeks on sham for both groups.
Dasilva et al., 2012	RCT, sham-controlled	Anodal tDCS	CM (*n* = 8).	10 sessions of 2 mA anodal for 20 min two or three times a week.	4 weeks	Contralateral-to-pain M1.	Reduced pain intensity.	Significant clinical improvement after 4 months.
Andrade et al., 2017	RCT, double blind, sham-controlled.	Anodal tDCS	CM (*n* = 13): 6 with M1 tDCS, 3 with DLPFC tDCS 4 in the sham	12 sessions of 2 mA lasting 20 min, three times a week.	4 weeks	M1, Left DLPFC.	Reduced Hit-6 score. Reduced pain.	Higher side effects in M1 group.
Silberstein et al., 2016	RCT, double blind, sham-controlled + open label phase months.	Neck VNS.	CM (*n* = 59).	Two unilateral 90 s doses three times a day.	2 months.	Vagus nerve at the neck.	Reduction in headache days in open label.	Phase 1 study.
Straube et al., 2015	RCT, double blind, sham-controlled.	Auricular VNS.	CM (*n* = 46), included MOH.	Active (25 Hz) or sham (1 Hz) stimulation.	4-h daily for 3 months.	Auricular branch of the vagus nerve.	Reduction in headache days in the sham arm.	
Di Fiore et al., 2017	Open-label study.	tSNS.	CM (*n* = 23), included MOH.	Build-up stimulation.	20 min/day for 4 months.		>50% reduction of headache days.	

*The results table present benefit obtained by single studies as reported by the original source. Due to differences in methodology and outcome measure considered, a direct comparison among studies is not possible. sTMS, single pulse TMS; rTMS, repeated pulse TMS; cTBS, continuous theta burst; tDCS, transcranial direct current stimulation; MA, migraine with aura; MoA, migraine without aura; CM, chronic migraine; MOH, Medication Overuse Headache; MT, motor threshold; tSNS, Transcutaneous supraorbital neurostimulation; vNS, vagus nerve stimulation; DLPFC, dorsolateral prefrontal cortex; M1, primary motor cortex; RCT, randomized controlled trial*.

These results in prevention are not easy to explain. sTMS was in fact tested in a pilot and later sham-controlled RCT (Clarke et al., 2006; Lipton et al., 2010). In this RCT, 164 subjects with episodic migraine (EM) self-administered sTMS over the occipital cortex during the aura phase or the beginning of an attack: 2-h pain free response rates were significantly higher with sTMS (39%) respect to sham stimulation (22%); treatment with sTMS showed a therapeutic gain of 17%.

The rational of the study was acting directly on migraine aura neural correlate, the cortical spreading depression, to abort the attack. So it is not clear how it can also prevent the repetition of new attacks. Two possible explanations led to the development of the ESPOUSE trial. One could be that several drugs used in migraine prevention inhibit CSD and, therefore, CSD inhibition can also be preventive of new attacks, alternatively, a modulation of the thalamic function induced by sTMS produced the prophylactic effect. Thalamus has a role in both attack development and central sensitization (Burstein et al., 2010). Beside that, however, we may think that sTMS may act in migraine prevention with an indirect mechanism. Repetition of the attack is in fact one of the main cause of chronification process. While painkillers or triptans drug therapies abuse facilitates central sensitization (clinically known as MOH), to date there is no evidence that the acute treatment of attack with sTMS induce sensitization producing a sort of “stimulation overuse headache”. We could therefore hypothesize that reducing the days of headache *per se* with neuromodulation has a prophylactic value against future attacks.

Repetitive TMS has also been studied in migraine prophylaxis, with conflicting results depending on the type (high vs. low frequency) and area of stimulation. The first study to evaluate rTMS in migraine was a pilot trial by Brighina and colleagues, in which six CM patients received 400 pulses of high-frequency (20 Hz) rTMS to the area corresponding to the dorso-lateral prefrontal cortex (DLPFC); five subjects received sham stimulation instead (Brighina et al., 2004). The 12 total stimulation sessions significantly reduced migraine attacks, as well as disability and use of abortive medication, respect to baseline. Significant differences in outcome measures were not observed in the placebo group. However, these results were not confirmed in a subsequent study in 18 migraine patients (9), who received a similar protocol of 1600 pulses 10 Hz stimulation over the DLPFC per session, for 23 sessions. After 8 weeks of treatment, the number of headache days decreased significantly more in the sham group than in the active rTMS-DLPFC group (Conforto et al., 2014).

Repetitive TMS applied over the primary motor cortex was also investigated in migraine, in a RCT of 100 episodic or CM patients. In this study, rTMS as preventive treatment was given in sessions of 600 pulses at 10 Hz on alternate days (Misra et al., 2013). The treatment was capable of significantly reducing headache frequency (from 78.7 to 33.3%) respect to placebo. Another study on 29 total CM patients compared the effects of rTMS over the motor cortex versus botulinum toxin-A injections (Shehata et al., 2016). The protocol was designed to deliver 20 trains of 100 stimuli at 10 Hz in tri-weekly sessions over 1 month. The treatment showed a reduction in headache frequency and a comparable efficacy to Botox, with, however, a less sustained effect. In a randomized trial using add-on deep rTMS vs. standard treatment, treatment-resistant CM patients received 10 Hz trains of 600 pulses in lateral and medial part of the prefrontal cortex bilaterally (according to authors the stimulation should reach DLPRF and orbitofrontal cortex). The 4 weeks period produced a decrement in pain intensity, number of headache days and also depressive symptoms compared to the pharmacological group (Rapinesi et al., 2016).

The biological rationale for the use of rTMS as a preventive treatment for migraine originates from the hypothesis of an abnormal cortical excitability of the migraineous brain. Repetitive TMS, with its effects on cortical depolarization and neuronal plasticity, could potentially repair this abnormal excitability in migraineurs. In an RCT by Teepker et al. (2010), the effects of low frequency rTMS in migraine prophylaxis were studied, based on the hypothesis of hyperexcitability in the migraineous brain. Interestingly the study, in which 27 migraneurs received 500 pulses of 1 Hz stimulation over the vertex, failed to show a significant decrease in headache frequency respect to sham stimulation (Teepker et al., 2010).

In a proof of concept studies, the efficacy of rTMS quadripulse applied over the visual cortex has recently been completed, however, results are still not available. The trial has been preceded by a proof of concept study for CM prevention (Sasso D’Elia et al., 2012), which showed a ≥50% reduction of migraine days in 40% of the 12 total participants.

The usefulness of modifying habituation deficit was partially confirmed by a recent study showing that active rTMS stimulation was capable of reducing the habituation deficit, measured through somatosensory evoked potentials, in 56 migraineurs; furthermore this normalization correlated with a parallel reduction in headache severity following 1 month of treatment (Kalita et al., 2017).

To date, one open-label clinical trial with cTBS has been implemented in migraine patients. It included both episodic (*n* = 6) and chronic patients (*n* = 3). The cTBS treatment improved the baseline by a 29% of total headache days immediately after the end of the stimulation and by -35% in the 4 weeks follow-up. Similarly, it reduced migraine attacks by 66% by the end of the treatment and by 88% 4 weeks later (Chen et al., 2016). Since no subanalysis is provided, drawing a firm conclusion in not possible.

#### Non-invasive Transcranial Direct Current Stimulation (tDCS)

To date only few studies selectively investigated the role of tDCS in CM (Dasilva et al., 2012; Rocha et al., 2015; Andrade et al., 2017) (see Table 2). In some other studies, chronic patients were recruited together with episodic, so that drawing a firm conclusion in not possible in the absence of a separate subanalysis (Antal et al., 2011; Rocha et al., 2015).

The first tDCS trial including CM patients was that performed by Antal et al. (2011). This was a randomized sham-controlled trial with crossover design. Out of the 30 patients enrolled, 26 participants complete the protocol and were included into the analysis. According to the hypothesis of hyperexcitability of the visual cortex, authors applied an inhibitory stimulation on the occipital cortex. Intensity of the active stimulation was 1 mA for 15 min every 2nd day for 3 weeks. Trial’s results were almost negative: neither active nor sham stimulation provided an improvement in primary endpoint (migraine attacks). Although active stimulation improved migraine-related days (-42.5%), mean duration of attacks (-19.5%) and intensity of pain (-22.6%), only the latter barely differed significantly from the sham treatment (*p* = 0.05).

The second RCT by Dasilva et al. (2012) included ten sessions over a 4 weeks period of anodal (or sham) tDCS over contralateral-to-pain M1 (Dasilva et al., 2012). Stimulation protocol used 2 mA for 20 min two or three times a week. This trial was aimed only to CM patients and recruited 13 patients distributed in a non-crossover design with 5 patients enrolled in the sham and 8 to the active group. The outcome measure was the reduction in pain. Although the difference between active and sham group was only close to significance immediately after the stimulation, active group showed clinical benefit overtime and significance difference was found after 4 months (-36.96%) and a trend for reduction in length of migraine episodes (in hours) of 88.75%. In this study, the sample size is quite small, and self-reported questionnaire and not a headache diary have been used to assess pain scores.

In a recent three arm study, one arm (M1-a) received active anodal stimulation aimed on left primary motor (M1), a second arm (DLPFC-a) received anodal tDCS on left dorsolateral prefrontal cortex (DLPFC) and a sham arm (SHAM-a) received sham stimulation also on left primary motor (M1) (Andrade et al., 2017). Thirteen CM patients were distributed in the three groups: 6 in the M1-a, 3 in the DLPFC-a, 4 in the SHAM-a. Stimulation protocol involved 12 sessions of 2 mA lasting 20 min, three times a week for 4 weeks. tDCS on DLPFC was more effective than M1 and sham stimulation. Direct comparison between M1 and DLPFC is lacking in the paper, except for the fact that M1 stimulation was associated to a higher risk of side effects (namely: headache, burning, and sleepiness).

### Peripheral Nerve Stimulations

#### Non-invasive Vagus Nerve Stimulation (nVNS)

The initial use of vagus nerve stimulation to treat headaches first came from the epilepsy field, following several anecdotal reports of migraine improvement in patients with comorbid epilepsy who had been implanted with the device (Sadler et al., 2002; Hord et al., 2003).

The breakthrough for its use in migraine therapy certainly came with the development of portable devices, which allow to stimulate the vagus nerve transcutaneously at the neck (GammaCore^®^ device) or in its auricular portion (Nemos^®^ device) in a non-invasive way.

The hypothesis for the effect of vagus nerve stimulation in headache lies on the presence of distinct anatomical and functional connections between the vagus nerve and the trigeminal complex (Kaube et al., 1993; Ruggiero et al., 2000). Furthermore, animal evidence has shown that vagus stimulation can reduce neuronal activity and glutamate levels in the spinal trigeminal nucleus, as well as pain (Ren et al., 1989; Randich et al., 1990; Lyubashina et al., 2012) and allodynia (Oshinsky et al., 2014) in the trigeminal area. This evidence overall seems to point to a nociceptive ascending modulating effect of the vagus nerve on the trigeminal system.

The GammaCore^®^ device was initially trialed for acute migraine therapy. In a first pilot study on 30 episodic migraine patients (27 of which entered the final analysis) 80 total attacks were treated with two right-sided 90 seconds sessions. A total of 22% of patients were pain free from moderate/severe attacks at 2 h, and 43% had pain relief at 2 h; 38% of the milder attacks were resolved at 2 h (Goadsby et al., 2014). Barbanti et al. (2015) administered the GammaCore^®^ device acutely in two unilateral 120 s doses in 48 patients; 14 subjects had high frequency episodic migraine and 36 CM. Results on 131 treated attacks showed a 39.6% pain free and 64.6% pain relief rate at 2 h from treatment (Barbanti et al., 2015). Side effects in both studies were transient and mild.

In the preventive setting, the GammaCore^®^ device has been used in a limited number of studies and it has to date not shown similar encouraging effects. A recent double-blind, sham-controlled RCT was performed on 59 CM patients who were treated with two unilateral 90 s doses three times a day for 2 months, and subsequently for an open label phase lasting up to 6 months (Silberstein et al., 2016). Outcomes were not significantly different between the sham and active stimulation group; however, at the end of the open label phase, the group initially assigned to nVNS - i.e., in the randomized phase- showed a significant reduction in headache days respect to baseline.

The Nemos^®^ device, developed in Germany, is used to stimulate the auricular branch of the vagus nerve through an electrode worn in the ear. In a recent RCT the efficacy of the device for preventive use was tested in 46 chronic migraineurs. Treatment was given in 4-h daily sessions with either active (25 Hz) or sham (1 Hz) stimulation (Straube et al., 2015). Results from this study were, however, disappointing, showing that subjects in the sham arm had a higher reduction in headache days than the ones receiving active stimulation.

#### Transcutaneous Supraorbital/Occipital Electrical Neurostimulation (tSNS and tONS)

Although it has been applied with clinical benefit in prevention of episodic forms, a clinical benefit from transcutaneous supraorbital electrical neurostimulation (tSNS) in CM is not established yet, although a clinical trial is currently ongoing (ClinicalTrials.gov identifier: NCT02342743). Small, open-label study showed that half of CM patients involved in the study had a reduction superior to 50% of the baseline number of headache days (Di Fiore et al., 2017).

Although the exact mechanism of action is not completely understood, some hints may come from one FDG-PET study on migraineurs that showed the effects of a 4-weeks long treatment with transcutaneous electrical neurostimulation (Magis et al., 2017).

In a sample of 10 subjects with migraine without aura, pretreatment FDG-PET showed a marked hypometabolism in the anterior cingulate cortex (ACC) and orbitofrontal cortex (OFC). At the 3 weeks follow-up, after tSNS treatment, patients reported at the group level a clinical benefit. The follow-up FDG-PET showed normalization in glucose metabolism in ACC and OFC. This change could be due either to stimulation effect or patients’ clinical improvement. However, some data points toward a slow neuromodulatory effect exerted by tSNS rather than to clinical improvement itself. The major fact in this direction is this increase didn’t differ between responders and not responders, so that a direct connection to clinical improvement seems relatively unlikely. One limit of this reasoning is that the sample size of the study was quite small, so that lack of difference may derive from low statistical power (Magis et al., 2017; Russo et al., 2017). However, both baseline hypometabolism of prefrontal cortices and their increase and after therapy increase were supported by other studies on neurostimulation in migraine (Matharu et al., 2004), cluster headache (Magis et al., 2011), or trigeminal neuropathic pain (Willoch et al., 2003).

## Protocol Indications, Nuances and Future Perspective

We have briefly reviewed the evidence supporting the idea that synaptic and anatomical, plasticity causes the state of hyperexcitability in CM. Both clinical (allodynia) and neurophysiological (pattern indistinguishable from the one found in ictal phase of episodic migraines) are in line with this interpretation.

For this reason, techniques of neurostimulation, which can modify in a predictable manner the thalamocortical interplay and, at the same time, induce plasticity and metaplasticity processes in neurons, are of primary importance in the treatment of migraine and especially CM. We know that cortical stimulation by tDCS and TMS can influence cortical and corticothalamic circuits and single pulse TMS also blocks the nociceptive neurotransmission from the thalamus to the cortex (Andreou et al., 2016; Sankarasubramanian et al., 2017).

In brief, what we can do with neurostimulation is

(1) increase or decrease cortical excitability in a target regions;
(2) modulate the interhemispheric and intrahemispheric functional connectivity by acting on functional connected brain areas in a facilitatory or inhibitory way;
(3) modulate the effect of a subsequent NIBS treatment by previously inducing LTP-like plasticity by means of a priming NIBS stimulation.

However, despite this large choice of stimulation, to date no clear indications have pointed out from the therapeutic studies performed until now, so that neither rTMS nor tDCS received any recommendation for use in migraine, except for the sTMS that is supported National Institute for Health and Clinical Excellence (NICE) in the United Kingdom for acute treatment (Lefaucheur et al., 2014, 2017). Several reasons account for that result.

In first place, some issues with therapeutic neuromodulation in CM are intrinsically related to the method. Some of them have been addressed in a recent paper by (Thibaut et al., 2017), where they deeply analyze some reasons why neuromodulation may fail. One point that they raised is very interesting because it fully influences some of the CM neuromodulation trials in this review: the intensity-related effect. In fact, previous studies showed that cathodal tDCS on the left motor cortex may have inhibitory effects when delivered at 1 mA, while excitatory effects when delivered at 2 mA (Batsikadze et al., 2008). In the last 5 years, safety limitations of tDCS changed and the maximum applicable limit passed from 1 to 2 mA. For this reason, some of the older trial, like (Antal et al., 2011), used cathodal stimulation on visual cortex at 1 mA to inhibit supposed hyperexcitability, while more recent trials, like (Rocha et al., 2015), used 2 mA stimulation for the same purpose. In this latter trial, the cathodal stimulation had no effect on phosphenes threshold that was used as neurophysiological measure. In the former trial, no neurophysiological measurement was used.

The second major point is that most of trials recruited small number of patients, so that they are generally underpowered. Not all of them, however, provided any neurophysiological surrogate marker of response beyond clinical improvement. On one hand, the response in migraine is only based on anamnestic recall and diary aid and, in CM patients with higher number of headache days, slight changes can go unnoticed. On the other hand in case of response the exact neurophysiological mechanism remains only speculative. Moreover some recent trial showed that neurophysiological modifications could also precede the clinical improvement suggesting how it is achieved (Viganò et al., 2018).

Another critical point is the choice of the clinical outcome measure. Some of the trials considered various combinations of pain intensity, attack frequency, headache days, and medication intake. This is problematic for two reasons. First, it does not allow comparing all trials easily. To over come this problem the International Headache Society released the updated guidelines for pharmacological and non-pharmacological controlled trials in episodic and CM (Silberstein et al., 2008).

Patient’s choice is fundamental in such trial. Clinically and neurophysiologically, CM patients differ from EM patients, and they should be kept separated in clinical trials. Some of the trials presented included both EM and CM patients, without better definition or subgroup analysis. By the same token, also patient with MOH should be object of different trials or at least subanalysis, especially when neurophysiological outcomes are considered since we know that some excitability indexes as sensitization and habituation vary in MOH vs. pure CM patients, and moreover, with the category of MOH, amongst triptans and analgesic overusers (Coppola et al., 2010).

In conclusion, at present, the major limitation of therapeutic neuromodulation studies is that only few studies also provided information on neurophysiological correlates produced by the stimulation and in some cases the clinical benefit was not associated to evident changes in neurophysiological parameters. In this line, it seems promising that targeting habituation deficit produced some reproducible results in episodic migraineurs (Viganò et al., 2013; Cortese et al., 2017b), however, it was not true at present for CM patients (Sasso D’Elia et al., 2012).

Further study, combining therapeutic and neurophysiological investigations (also aimed to investigate plasticity changes) are then needed to better understand the complexity of NIBS restorative effects and define the better therapeutic interventions.

## Author Contributions

AV and MT conceived the idea and the topic. AV provided the introduction, the part on displasticity in chronic migraine, and the part on TDCS and STS. MT provided the part on plasticity. FP provided the part on TMS and NVS in migraine. VDP supervised and re-edited the text. All authors drafted the final text.

## Conflict of Interest Statement

The authors declare that the research was conducted in the absence of any commercial or financial relationships that could be construed as a potential conflict of interest.
